# Antibiofilm Activity of Three Essential Oils Against ESBL-Producing *Klebsiella pneumoniae*: An In Vitro and In Silico Investigation of Putative Molecular Targets

**DOI:** 10.3390/antibiotics15070647

**Published:** 2026-06-29

**Authors:** Karim Bariz, Bilal Saoudi, Souad Lahcene, Idir Moualek, Hillal Sebbane, Fares Rekbi, Hakim Belkhalfa, Assia Derguini, Nasir A. Ibrahim, Sulaiman Abdullah Ali Alsalamah, Mohammed Saad Aleissa, Nosiba S. Basher, Lamia Trabelsi, Karim Houali

**Affiliations:** 1 Laboratory of Analytical Biochemistry and Biotechnology, Faculty of Biological and Agronomic Sciences, Mouloud MAMMERI University of Tizi Ouzou, Tizi Ouzou 15000, Algeria; karim.bariz@ummto.dz (K.B.); saoudi.bilal@fsbsa.ummto.dz (B.S.); souad.lahcen@ummto.dz (S.L.); idir.moualek@ummto.dz (I.M.); hilal.sebbane@ummto.dz (H.S.); karim.houali@ummto.dz (K.H.); 2 Research Centre in Industrial Technologies–CRTI, Cheraga P.O.Box 64, Algiers 16014, Algeria; f.rekbi@crti.dz; 3 Scientific and Technical Research Centre in Physico–Chemical Analysis (CRAPC), Ouaregla 30000, Algeria; hakimbelkhalfa@gmail.com; 4 Microbial Ecology Laboratory, Faculty of Natural and Life Sciences, Abderrahmane MIRA University, Bejaïa 06000, Algeria; assia.derguini@univ-bejaia.dz; 5 Biology Department, College of Science, Imam Mohammad Ibn Saud Islamic University (IMSIU), Riyadh 11623, Saudi Arabia; naabdalneim@imamu.edu.sa (N.A.I.); saaisalamah@imamu.edu.sa (S.A.A.A.); msaleissa@imamu.edu.sa (M.S.A.); 6 Marine Biodiversity Laboratory, National Institute of Marine Sciences and Technology (INSTM), University of Carthage, Tunis 1054, Tunisia; lamia.trabelsi@instm.rnrt.tn

**Keywords:** *Klebsiella pneumoniae*, multidrug resistance, biofilm, antibiofilm activity, essential oil, molecular docking

## Abstract

Biofilm formation is a major contributor to antibiotic resistance in *Klebsiella pneumoniae*, posing a serious challenge to current therapeutic strategies. Thus, this study aims to evaluate the antibiofilm activity of three essential oils *Thymus hirtus* Willd. Ssp. *algeriensis* Boiss, *Syzygiuma romaticum*, and *Eucalyptus globulus* against four clinical isolates of ESBL-producing *K. pneumoniae*, along with the reference strain *K. pneumoniae* ATCC 700603. The antibiofilm activity of essential oils was assessed with crystal violet assay using MICs ranging from 3.38 ± 0.2 to 27.1 ± 0.56 mg/mL, 2 ± 0.19 to 32 ± 0.55 mg/mL, and 13.78 ± 0.62 to 110.25 ± 3.37 mg/mL, for TEO, SEO and EEO, respectively. In vitro tests showed that *S. aromaticum* EO and *T. algeriensis* EO exhibited the best anti-adhesive activity with a percentage of up to 75.39%, while no difference was observed between the EO in their eradication activity. Microscopic observations confirmed the disorganization of the biofilm after treatment with *T. algeriensis*. The molecular docking analysis of the three EOs main compounds with MrkH, SdiA and MrkD revealed that SdiA was the most favorable target, with p-cymene (−7.7 kcal/mol), α-pinene (−7.5 kcal/mol), and eucalyptol (−7.1 kcal/mol) showing the strongest binding affinities. Thymol and p-cymene showed also a favorable affinity with MrkD. Overall, *p*-cymene and α-pinene demonstrated the most favorable binding profiles, whereas linalool exhibited the weakest predicted interactions. These results highlight the promising potential of these EOs, as multi-target antibiofilm agents against MDR- *K. pneumoniae* biofilms.

## 1. Introduction

*Klebsiella pneumoniae* is a Gram–negative bacillus belonging to the *Enterobacteriaceae* family and is recognized as a major opportunistic pathogen involved in a wide range of healthcare-associated infections. As a member of the ESKAPEE (*Enterococcus faecium*, *Staphylococcus aureus*, *K. pneumoniae*, *Acinetobacter baumannii*, *Pseudomonas aeruginosa*, *Enterobacter* spp., and *Escherichia coli*) group, *K. pneumoniae* is particularly concerning due to its capacity to acquire and disseminate multiple antibiotic resistance determinants [1]. Among these mechanisms, the production of extended-spectrum β-lactamases (ESBLs) represents a major clinical challenge, as ESBL-producing *K. pneumoniae* strains can hydrolyze a broad range of β-lactam antibiotics, including third-generation cephalosporins, and are frequently associated with therapeutic failure in hospital settings [2]. In recent years, resistance to last-resort antibiotics, including novel β-lactam/β-lactamase inhibitor combinations, has been increasingly reported, maintaining *K. pneumoniae* on the World Health Organization’s critical priority list [3,4].

One of the key contributors to antibiotic tolerance in *K. pneumoniae* is its ability to form biofilms, structured microbial communities embedded in a self-produced extracellular polymeric matrix that confers protection against host immune responses and antimicrobial agents. Cells within biofilms are 1000 times more resistant to antibiotics compared with their planktonic counterparts, due to the presence of thick EPS layers, enhanced expression of efflux pumps, and the presence of persistent cells [5,6,7]. Currently, most (65–80%) bacterial infections are associated with biofilm formation [7].

The formation and maintenance of biofilms are regulated by multiple proteins involved in adhesion, fimbrial expression, and quorum sensing (QS). Among these, Mannose-resistant Klebsiella-like H (MrkH) and Mannose resistant Klebsiella-like D (MrkD) play essential roles in type 3 fimbriae-mediated adhesion, while the Suppressor of cell division A (SdiA), a transcriptional regulator, contributes to intercellular signaling and the regulation of biofilm-associated genes [8,9,10]. Despite their crucial roles in biofilm establishment, these proteins have received limited attention as potential molecular targets for antibiofilm interventions.

Given the limited efficacy of conventional antibiotics against biofilm-associated infections, increasing attention has been directed toward natural products as complementary sources of antibiofilm agents. Among these, essential oils (EOs), complex mixtures of bioactive secondary metabolites, have been reported to exhibit antimicrobial and antibiofilm activities against various pathogenic bacteria [11,12,13]. In particular, EOs derived from *Eucalyptus globulus*, *Syzygium aromaticum*, and *Thymus hirtus* Willd. Ssp. *algeriensis* Boiss have demonstrated notable antimicrobial properties, making them promising candidates for further investigation against multidrug-resistant *K. pneumoniae*.

The essential oil of *E. globulus*, a species belonging to the *Myrtaceae* family, is rich in oxygenated monoterpenes, particularly 1,8-cineole, which has been associated with antibacterial activity and interference with bacterial cell integrity [14]. In addition, the antibiofilm potential of this EO has been highlighted against clinically relevant pathogens, including resistant *K. pneumoniae* isolates [15]. Similarly, *Syzygium aromaticum* (clove) essential oil is characterized by a high content of phenylpropanoids, mainly eugenol, which has been extensively studied for its antimicrobial and antibiofilm effects. Eugenol has been reported to disrupt biofilm formation and reduce established biofilms in carbapenemase and ESBL, producing *K. pneumoniae* strains, suggesting its potential role as an antibiofilm agent [16]. In contrast, *Thymus hirtus* Willd. Ssp. *algeriensis* Boiss, a thyme species endemic to North Africa, is known for its chemically diverse essential oil, typically rich in monoterpenes and phenolic compounds. While its antimicrobial activity against planktonic bacterial cells has been documented [17], information regarding its antibiofilm activity, particularly against multidrug-resistant *K. pneumoniae*, remains limited, highlighting a relevant knowledge gap.

Building on our previous work characterizing the chemical composition and antibacterial activity of these essential oils against planktonic *K. pneumoniae* cells [18], the present study aimed to evaluate their antibiofilm activity against ESBL-producing *K. pneumoniae*. In addition, a molecular docking approach was used to explore potential interactions between the main components of these essential oils and MrkH, MrkD, and SdiA, to provide mechanistic information for their relevance as potential antibiofilm targets in *K. pneumoniae*.

## 2. Results

### 2.1. Biofilm Formation Ability

The results of the biofilm formation assay are presented in Table 1. Based on the findings, it was observed that *K. pneumoniae* 1216 and 5111 exhibited weak and moderate biofilms formation, with an absorbance value of 0.103 ± 0.005 and 0.292 ± 0.021, respectively. Meanwhile, the other strains, including the reference strain, were all strong biofilm formers with absorbance values ranging from 0.292 ± 0.021 to 1.131 ± 0.058.

### 2.2. Anti-Adhesion Activity

**Figure 1:**
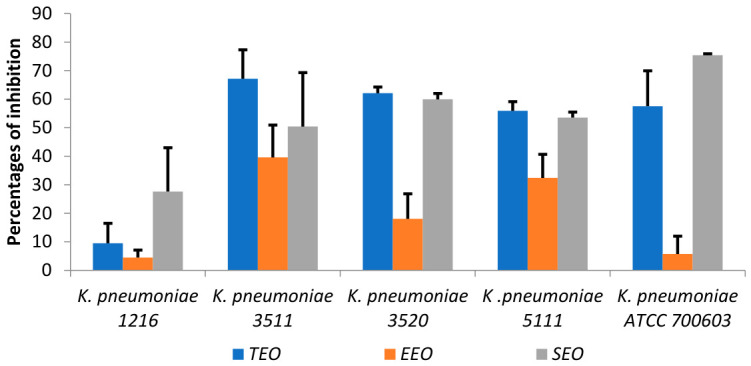
Inhibition Percentages of Biofilm Formation by the Three EOs.

The results of the antiadhesion activity and biofilm development inhibition of the three essential oils tested are shown in Figure 1. The inhibition percentages ranged from 9.52% to 67.17% for *Thymus hirtus* Willd. Ssp. *algeriensis* Boiss essential oil (TEO) against the five strains tested, while for *S. aromaticum* essential oil (SEO) and *E. globules* essential oil (EEO), the inhibition percentages ranged from 27.64% to 75.37% and 4.23% to 39.61%, respectively. The ANOVA statistical test revealed that the EEO activity differed significantly (*p* < 0.05) from both TEO and SEO, while no significant difference was detected between TEO and SEO.

### 2.3. Eradication of Preformed Biofilm

**Figure 2:**
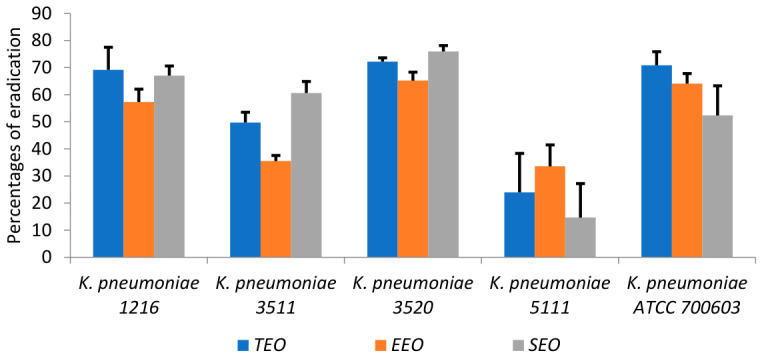
Percentages of biofilm eradication by the three tested EOs.

The effect of the three tested essential oils on biofilm eradication is shown in Figure 2. It appears that the percentages of biofilm eradication vary from 23.95% to 72.19% for TEO, from 14.68% to 75.96% for SEO, and from 33.56% to 65.20% for EEO. The ANOVA statistical test showed no significant difference (*p* > 0.05) in the eradication activity of the three oils.

### 2.4. Scanning Electron Microscopy (SEM)

**Figure 3:**
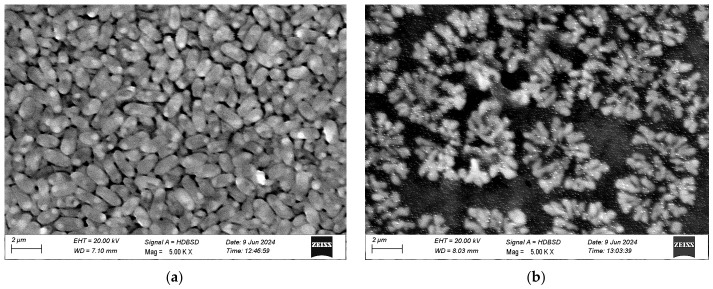
Scanning Electron Microscope Imaging of *K. Pneumoniae* ATCC 700603 biofilm: (**a**) untreated biofilm; (**b**) biofilm treated with TEO (MIC) for 60 min.

**Figure 4:**
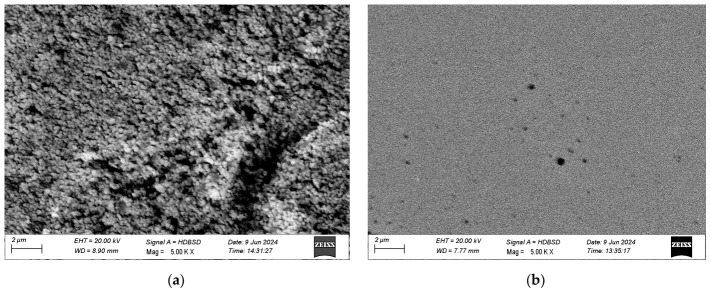
Scanning Electron Microscope Imaging of *K. Pneumoniae* ATCC 3520 biofilm: (**a**) untreated biofilm; (**b**) biofilm treated with TEO (MIC) for 60 min.

Observations made using a scanning electron microscope (GX 5000) (CIQTEK SEM5000X, Hefei, China) to observe the effect of TEO are shown in Figure 3 and Figure 4. The images of the positive control (Figure 3a and Figure 4a), namely the *K. pneumoniae* ATCC and *K. pneumoniae* 3520 strains, show a structured and organized biofilm for the reference strain with homogeneous cells, while that of the pathogenic strain *K. pneumoniae* 3520 appears denser, making the cells difficult to individualize. After 60 min of treatment of the biofilms with TEO, the results observed in Figure 3b and Figure 4b show a disorganization of the biofilm formed by the reference strain, accompanied by a change in cell morphology with the appearance of star-shaped dendrites, as well as a total eradication of the *K. pneumoniae* 3520 biofilm with the persistence of cellular debris from the biofilm formed.

### 2.5. Molecular Docking Analysis

A validation test of our procedure by redocking yielded an RMSD value < 2 A°, confirming the robustness of our method for exploring the interactions of our ligands of interest with the target proteins.

**Figure 5:**
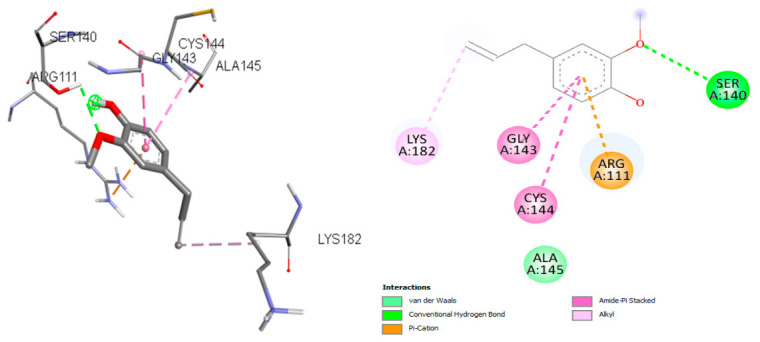
3D and 2D interaction representations of eugenol with MrkH.

**Figure 6:**
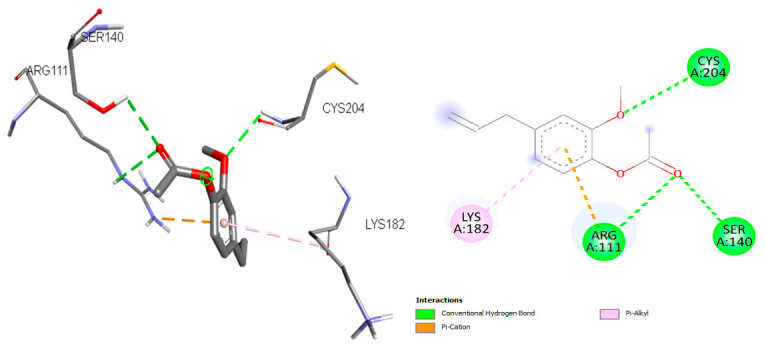
3D and 2D interaction representations of eugenol acetate with MrkH.

With regard to the MrkH regulator, the results reported in Table 2 and Figure 5 and Figure 6 showed that the two major compounds in *S. aromaticum* essential oil were able to form complexes with the protein, with free binding energies (ΔG) of −5.7 and −5.5 kcal/mol for eugenol and eugenol acetate, respectively. Eugenol formed five bonds: one of which was a conventional hydrogen bond with Ser140, an electrostatic bond with Arg111, three hydrophobic bonds with Gly143, Cys144, and Lys182, and a Van der Waals interaction with Ala 145. On the other hand, eugenol acetate showed three conventional hydrogen bonds with Ser140, Arg111, and Cys204, one electrostatic bond with Arg111, and another hydrophobic bond with Lys182.

**Figure 7:**
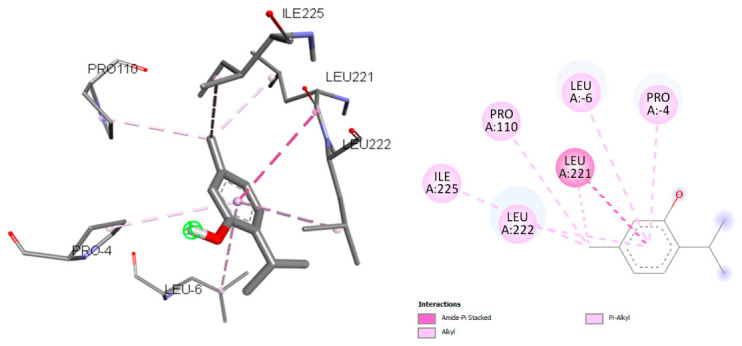
3D and 2D interaction representations of thymol with MrkH.

**Figure 8:**
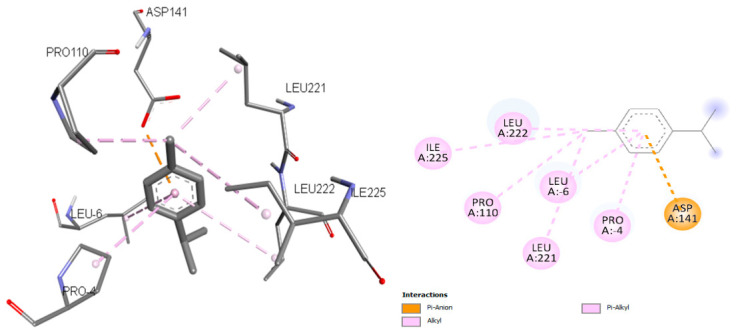
3D and 2D interaction representations of *p*-cymene with MrkH.

**Figure 9:**
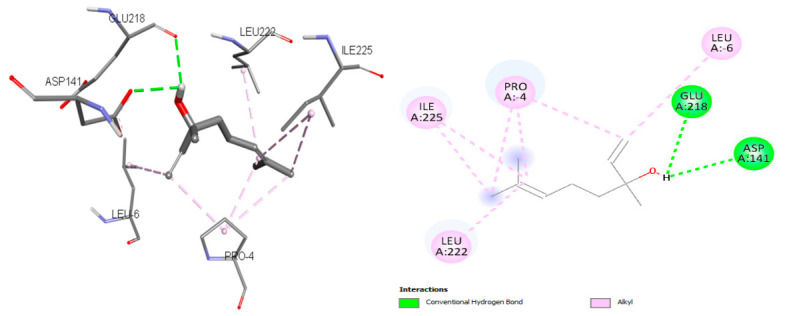
3D and 2D interaction representations of linalool with MrkH.

Concerning TEO, the three predominant compounds, thymol, *p*-cymene, and linalool interacted with MrkH protein with binding free energies of −5.0, −5.3, and −4.9 kcal/mol, respectively (Table 2). Thymol interacted exclusively through hydrophobic bonds with residues Leu221, Pro110, Ile225, Leu-6, Pro-4, and Leu222 (Figure 7). *p*-Cymene formed one electrostatic interaction with Asp141 and several hydrophobic interactions involving Pro110, Leu221, Ile225, Leu-6, Pro-4, and Leu222 (Figure 8). While linalool established two conventional hydrogen bonds with Asp141 and Glu218, in addition to multiple hydrophobic interactions with Pro-4, Leu222, Ile225, and Leu-6 (Figure 9).

**Figure 10:**
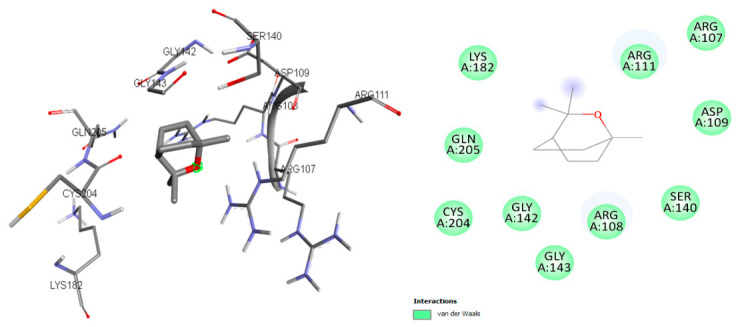
3D and 2D interaction representations of eucalyptol with MrkH.

**Figure 11:**
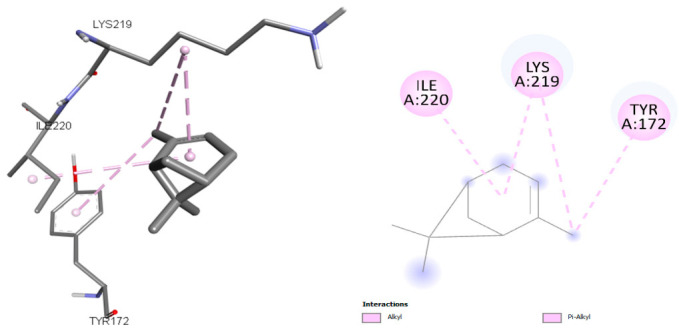
3D and 2D interaction representations of *α*-pinene with MrkH.

In the case of *E. globulus* EO compounds, eucalyptol bound to MrkH protein with a binding free energy of −4.7 kcal/mol (Table 2), through Van der Waals interactions with Arg107, Arg108, Arg109, Arg111, Ser140, Gly142, Gly143, Lys182, Cys204, and Gln205 (Figure 10). Whereas *α*-pinene exhibited a binding free energy of −4.6 kcal/mol (Table 2), by establishing hydrophobic interactions with Lys219, Ile220, and Tyr172 (Figure 11).

**Figure 12:**
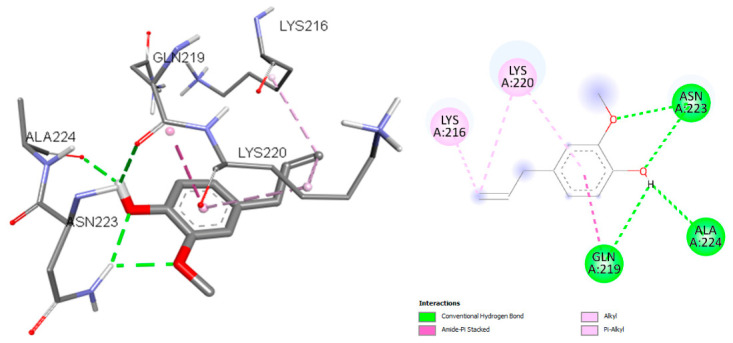
3D and 2D interaction representations of eugenol with SdiA.

**Figure 13:**
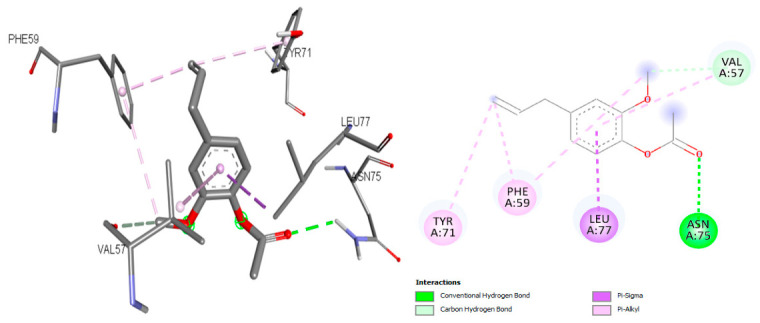
3D and 2D interaction representations of eugenol acetate with SdiA.

With regard to the SdiA protein, eugenol and eugenol acetate interacted with the target at different sites, with ΔG values of −4.3 and −5.5 Kcal/mol, respectively (Table 3). As shown in Figure 12, eugenol formed conventional hydrogen bonds with Asn223, Gln219, and Ala224, and hydrophobic bonds with Lys220, Gln219, and Lys216. While eugenol acetate bound to the protein with conventional and carbonyl hydrogen bonds involving Asn75 and Val57, respectively, as well as hydrophobic bonds targeting Leu77, Phe59, Tyr71, and Val57 (Figure 13).

**Figure 14:**
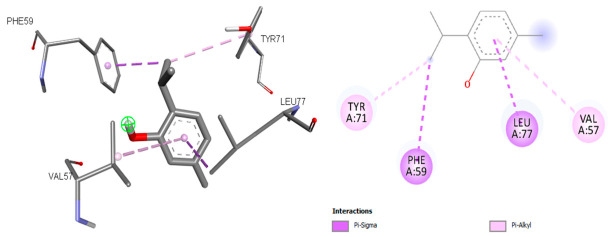
3D and 2D interaction representations of thymol with SdiA.

**Figure 15:**
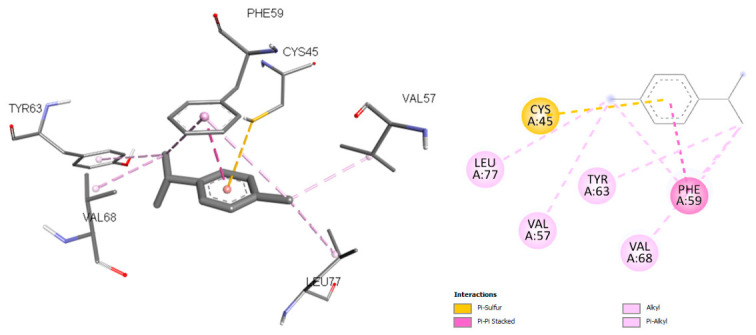
3D and 2D interaction representations of *p*-cymene with SdiA.

**Figure 16:**
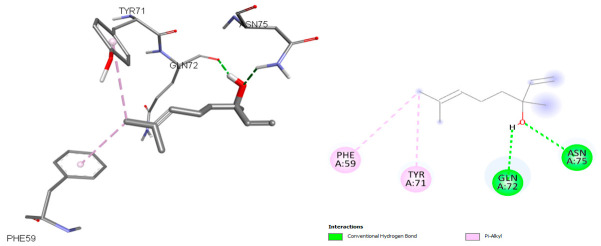
3D and 2D interaction representations of linalool with SdiA.

In the context of TEO, *p*-cymene exhibited the strongest binding affinity (−7.7 kcal/mol), followed by thymol (−5.7 kcal/mol) and linalool (−4.2 kcal/mol) (Table 3). Thymol interacted predominantly through hydrophobic bonds involving Leu77, Phe59, Tyr71, and Val57 (Figure 14). *p*-Cymene also formed hydrophobic interactions with Phe59, Val57, Val68, Leu77, and Tyr63, along with a π-sulfur interaction with Cys45 (Figure 15). In contrast, linalool established two conventional hydrogen bonds with Asn75 and Gln72, in addition to hydrophobic interactions with Phe59 and Tyr71 (Figure 16).

**Figure 17:**
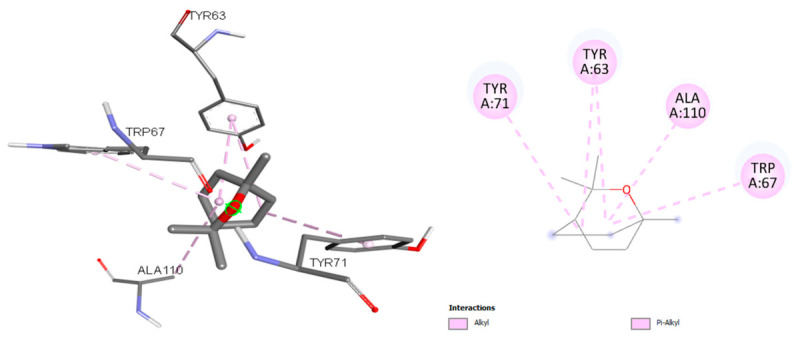
3D and 2D interaction representations of eucalyptol with SdiA.

**Figure 18:**
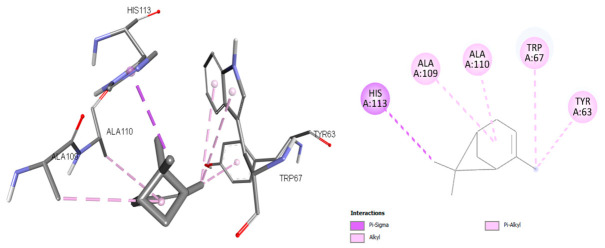
3D and 2D interaction representations of *α*-pinene with SdiA.

Docking analysis of EEO components targeting the SdiA protein revealed predicted binding free energies of −7.1 and −7.5 kcal/mol for eucalyptol and α-pinene, respectively (Table 3). Eucalyptol interacted mainly through hydrophobic interactions involving Ala110, Tyr63, Trp67, and Tyr71 (Figure 17). Similarly, *α*-pinene formed hydrophobic bonds with His113, Ala109, Ala110, Tyr63, and Trp67 (Figure 18).

**Figure 19:**
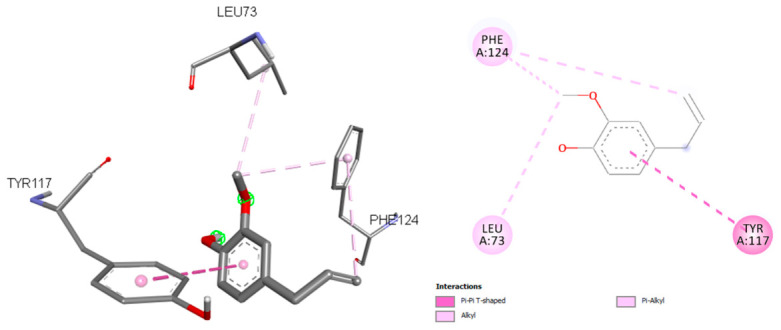
3D and 2D interaction representations of eugenol with MrkD.

**Figure 20:**
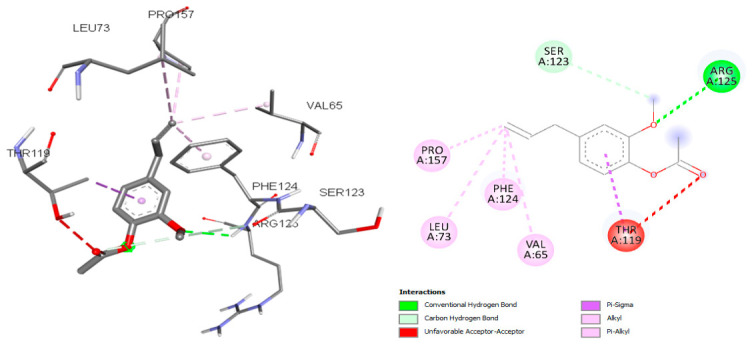
3D and 2D interaction representations of eugenolacetate with MrkD.

The results of the interaction of the major compounds of the three tested EOs with MrkD adhesin are reported in Table 4. The two main compounds of *S. aromaticum*, eugenol and eugenol acetate, interacted with the protein, with a predicted binding energy of −5.4 and −5.7 kcal/mol, respectively. Eugenol exhibited hydrophobic interactions with Leu73, Tyr117, and Phe124 (Figure 19). While eugenol acetate showed hydrogen bonds with Ser123 and Arg125, in addition to the hydrophobic bonds with Leu73, Val65, Phe124, and Pro157 (Figure 20).

**Figure 21:**
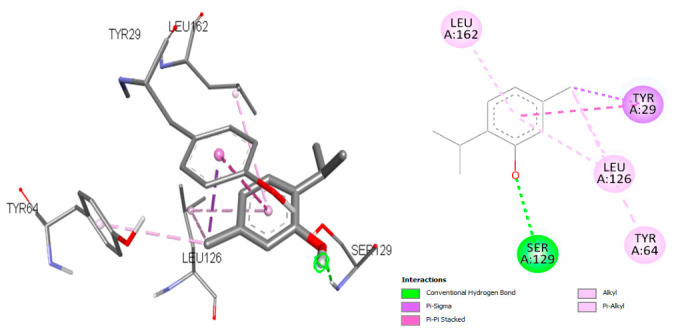
3D and 2D interaction representations of thymol with MrkD.

The three TEO compounds, thymol, *p*-cymene, and linalool, formed complexes with MrkD, with respective ΔG values of −7.0, −6.4, and −5.5 kcal/mol. Figure 21 shows that thymol binds to the protein with a conventional hydrogen bond with Ser129 and hydrophobic bonds with Thy29, Leu126, Thy64, and Leu162.

**Figure 22:**
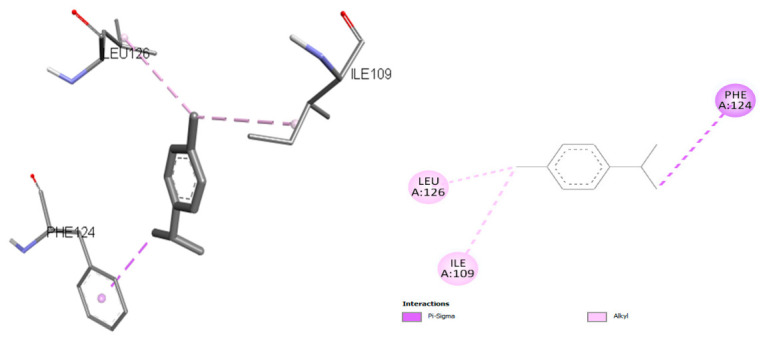
3D and 2D interaction representations of *p*-cymene with MrkD.

**Figure 23:**
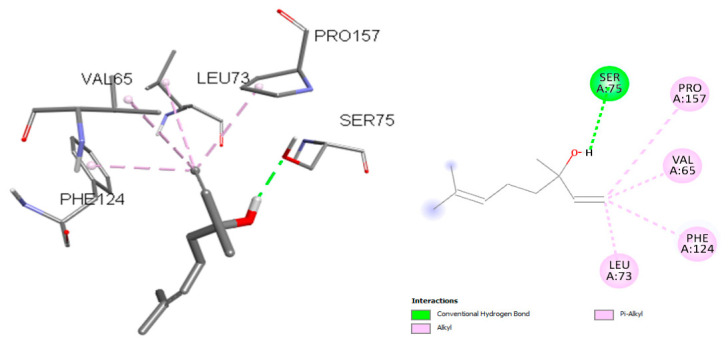
3D and 2D interaction representations of linalool with MrkD.

*p*-Cymene, formed only three hydrophobic bonds with Phe124, Ile109, and Leu126, which are shown in Figure 22. Finally, linalool interacted with MrkD through a conventional hydrogen bond with Ser75 and hydrophobic bonds with Val65, Leu73, Pro157, and Phe124 (Figure 23).

**Figure 24:**
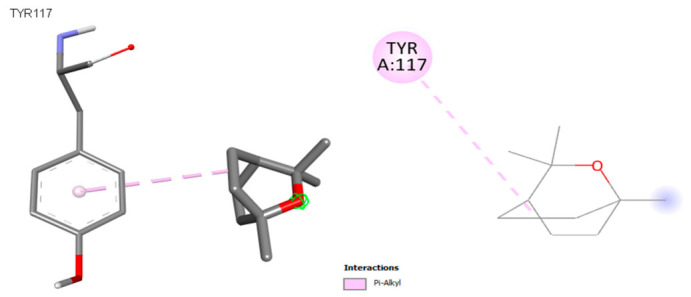
3D and 2D interaction representations of eucalyptol with MrkD.

**Figure 25:**
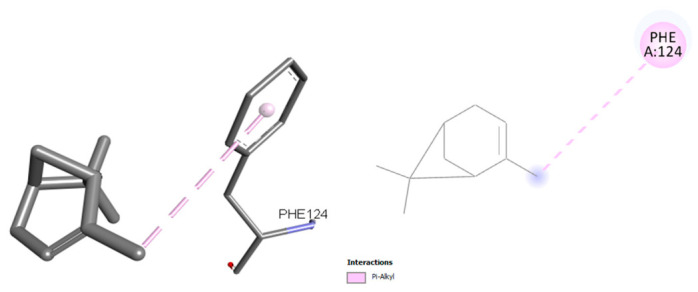
3D and 2D interaction representations of *α*-pinene with MrkD.

The analysis of EEO components interactions with MrkD showed that eucalyptol displayed a binding free energy of −5.3 kcal/mol (Table 4), forming a hydrophobic interaction with Tyr117 (Figure 24). In comparison, α-pinene demonstrated a score of −6.0 kcal/mol (Table 4) by establishing a hydrophobic bond with Phe124 (Figure 25).

**Figure 26:**
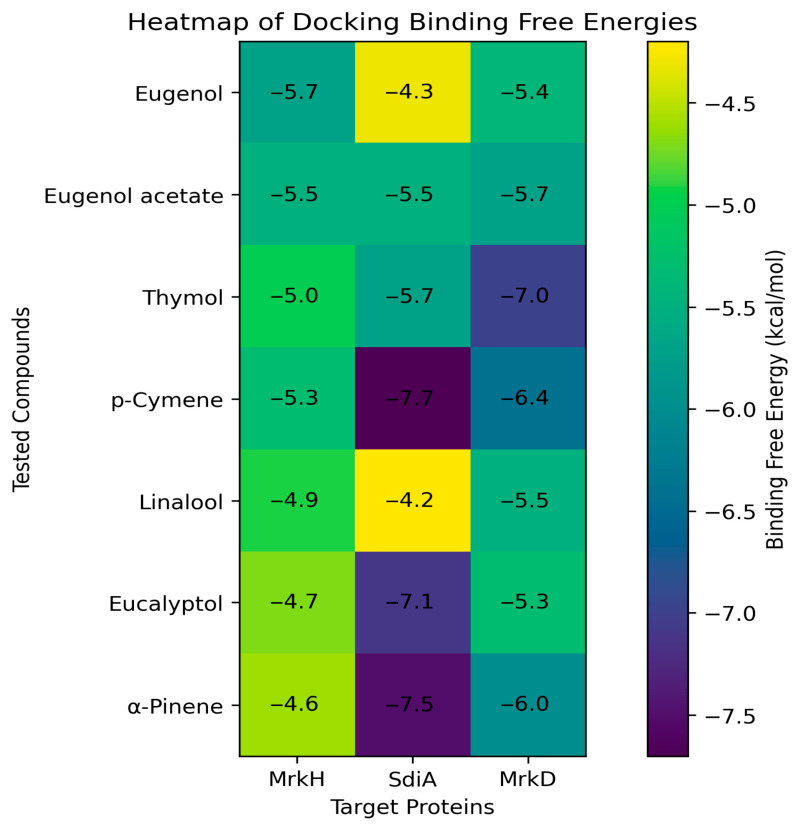
Heatmap of molecular docking scores of essential oils components.

In order to provide a clearer comparative overview of the binding affinities, the ΔG values were additionally represented as a heatmap (Figure 26), allowing visualization of the relative performance of each compound across the three protein targets.

Based on the overall docking scores across the three targets, the best binding energy was observed for the *p*-cymene-SdiA complex (−7.7 kcal/mol), followed by α-pinene-SdiA (−7.5 kcal/mol) and eucalyptol–SdiA (−7.1 kcal/mol). Thymol-MrkD and *p*-cymene-MrkD also demonstrated favorable binding energies of −7.0 and −6.4 kcal/mol, respectively. Moderate affinities were observed for *α*-pinene–MrkD (−6.0 kcal/mol), eugenol acetate-MrkD (−5.7 kcal/mol), and thymol–SdiA (−5.7 kcal/mol). While theremaining complexes exhibited binding free energies ranging between −5.5 and −4.2 kcal/mol, indicating comparatively weaker predicted interactions.

When considering the overall performance of the tested ligands, *p*-cymene and *α*-pinene emerged as the most favorable compounds, followed by thymol and eucalyptol. Eugenol acetate and eugenol displayed moderate binding profiles, whereas linalool showed the lowest overall docking performance.

## 3. Discussion

Antibiotic resistance makes biofilm-associated infections particularly worrisome due to their complex microbial structure, which greatly hinders the effectiveness of antimicrobial treatments. Indeed, biofilms consist of organized bacterial communities protected by an extracellular matrix rich in proteins, polysaccharides, and genetic material of bacterial and sometimes host origin [19]. This organization gives bacteria increased resistance not only to antibiotics but also to immune defenses, particularly the complement system, antimicrobial peptides, and phagocytosis.

In this study, most ESBL-producing *Klebsiella pneumoniae* isolates exhibited a strong biofilm-forming phenotype, with limited variability among strains. This finding is consistent with previous reports describing a high biofilm-forming capacity among multidrug-resistant *K. pneumoniae* [20,21,22,23,24]. The pronounced biofilm production observed in the clinical isolates from Tizi-Ouzou University Hospital may contribute to their persistence in the hospital environment and to therapeutic failure. Indeed, biofilm-associated cells display significantly increased resistance to antibiotics compared to their planktonic counterparts and may act as reservoirs for the dissemination of ESBL genes [25]. These observations further support the need to explore alternative strategies targeting biofilm-related virulence mechanisms.

In this context, the present study aimed to evaluate the antibiofilm potential of the essential oils of *Thymus hirtus* Willd. Ssp. *algeriensis* Boiss, *S. aromaticum*, and *E. globulus*. The selection of these three plants was guided primarily by their availability in our region, their broad traditional medicinal use, and their satisfactory essential oil yields, supporting their potential for local therapeutic valorization. Moreover, although botanically unrelated, they were intentionally chosen to represent chemically diverse EOs, allowing a broader exploration of phytochemical interactions with biofilm-associated targets.

Our previous study showed that TEO, SEO, and EEO had MICs ranging from 3.38 ± 0.2 to 27.1 ± 0.56 mg/mL, 2 ± 0.19 to 32 ± 0.55 mg/mL, and 13.78 ± 0.62 to 110.25 ± 3.37 mg/mL, respectively, against these MDR isolates of *K. pneumoniae* [18]. The evaluation of antibiofilm activity, using concentrations equivalent to MICs, revealed that these three EOs exhibited varying degrees of activity against these strains. Clove and thyme essential oils showed the strongest anti-adhesion activity, whereas EEO showed comparatively lower efficacy. These findings are consistent with previous reports demonstrating the antibiofilm potential of phenolic-rich EOs, particularly those containing thymol and eugenol. Indeed, Qian et al. [26] demonstrated that eugenol, the main constituent of clove EO, inhibited the initial stage of biofilm formation in carbapenemase-producing *K. pneumoniae* strains. Poojara et al. [27] showed that clove EO and eugenol could block the biofilm formation in *Vibrio cholerae* O1 strains, at the adhesion phase.

Regarding the thyme EO, Dizani and Asadpour [28] reported that thymol inhibited biofilm formation in *K. pneumoniae* strains isolated from hospitals in Iran. Furthermore, concerning its ability to eradicate biofilm, Bouguenoun et al. [29] demonstrated the complete eradication of biofilms in five *K. pneumoniae* strains using *T. pallenscens* EO at a concentration of 10 mg/mL, and Abdelhamed et al. [30] revealed total eradication of *K. pneumoniae* biofilm by *T. vulgaris* EO. Furthermore, the reduction in cell number, the disorganization of the biofilm, and the decrease in cell aggregation observed by SEM in our study were also reported by Yao et al. [31] on clinical *K. pneumoniae* biofilms exposed to thymol. Similarly, Alavi and Karimi [32] reported a change in cell morphology and the appearance of star-shaped dendrites on a biofilm of a clinical strain *K. pneumonia* ESBL-positive, following treatment of its biofilm with thymol.

With regard to *E. globulus* EO, several studies have reported that despite its antimicrobial activity, this oil is often less effective against biofilms than oils rich in phenolic compounds [33,34,35]. Indeed, according to Merghni et al. [36], the action of eucalyptus is often limited to the initial stages of adhesion or inhibition of bacterial metabolism, without profoundly disrupting the established biofilm, unlike phytophenols (thymol, eugenol), which can disrupt matrix cohesion and adhesive interactions. Taken together, these findings suggest that differences in antibiofilm efficacy among the tested essential oils may be associated with variations in their chemical composition.

In order to gain deeper insight into the molecular basis underlying these differences and to investigate the possible mechanisms involved in the antibiofilm activity of the tested EOs, a molecular docking analysis was performed. This is a computational approach used to predict and analyze the interactions between a ligand and a target protein. It estimates the preferred binding orientation within the active site and evaluates the strength of the interaction through a scoring function expressed as binding free energy (ΔG), where more negative values indicate greater binding stability. This method is widely applied in structure-based drug design for the in silico identification of potential bioactive compounds [37,38].

In this study, it was hypothesized that the inhibitory effect of the tested EOs on *K. pneumoniae* biofilm formation could be attributed to the ability of their main compounds to interact with proteins that regulate biofilm formation, such as MrkH and SdiA. In contrast, the eradicating potential could be explained by the interaction of these major components with surface proteins that ensure bacterial adhesion, like MrkD adhesin. Therefore, the choice of these proteins as targets for docking can be justified by their crucial involvement in the formation and maintenance of biofilm in this bacterium.

MrkH is a key transcriptional regulator in *K. pneumoniae*, responsible for activating the *mrk*ABCDF operon, which encodes the subunits of type 3 fimbriae [8]. This protein functions through allosteric activation by c-di-GMP, which binds to a PilZ domain consisting of two conserved motifs, the first of which contains an arginine chain (residues 109 to 113), and the second of which extends from residue 140 to 145 [39]. In our study, docking of MrkH with the main EOs components revealed the formation of complexes with moderate binding affinities (ΔG values from −5.7 to −4.6 kcal/mol). The two main SEO compounds showed the best affinities, followed by those of TEO and EEO, which are in line with their in vitro activities. These differences appear to be strongly influenced by the structural features and functional groups of each molecule. Indeed, the strongest binding affinity exhibited by eugenol and eugenol acetate could be attributed to the phenolic—OH, which is particularly important to enhance ligand-protein stability complex through hydrogen bonds, in addition to hydrophobic interactions of the aromatic ring [40]. Thymol and p-cymene interacted mainly through hydrophobic contacts. However, thymol contains a phenolic hydroxyl group; no hydrogen bonds were observed, which could be explained by the unfavorable orientation of the molecule in the binding pocket, preventing optimal geometric alignment with suitable hydrogen-bond donors or acceptors [41]. On the other hand, linalool showed lower affinity despite its hydrogen bonds, which could be attributed to the absence of an aromatic ring, making it more flexible and thus reducing binding stability due to entropic penalties upon binding [42]. The two major components of EEO (Eucalyptol and α-pinene) exhibited the lowest binding scores, which could result from the predominance of weak Van der Waals (Eucalyptol) and limited hydrophobic interactions (α-pinene), combined with the absence of polar anchoring groups capable of forming stabilizing hydrogen bonds or electrostatic contacts. Most of these interactions involved several key residues of the Pilz domain, such as Arg111, Ser140, Gly143, Cys144, and Ala145, suggesting that the tested EOs compounds could interfere with the binding of c-di-GMP to MrkH, thereby preventing activation of the *mrk* operon, which potentially reduces the expression of type 3 fimbriae and the bacterium’s ability to adhere and form biofilm. To our knowledge, no previous study has targeted this protein using a docking approach. However, some reports have shown that eugenol is capable of interacting with pilus assembly proteins, such as Csu C and Csu E, in *Acinetobacter baumannii*, which supports the hypothesis of its indirect antibiofilm effect by disrupting the formation of adhesion structures [43,44].

The SdiA protein is a transcriptional regulator that controls multiple processes, including cell division and the expression of virulence factors such as fimbriae, and curli-encoding genes. Pacheco et al. [10] highlighted its importance in biofilm formation by observing that SdiA, deficient strains showed a reduced expression of type 3 fimbriae genes, suggesting that it could be a promising target against biofilm formation. Functionally, this regulator is capable of acting both in the absence and presence of N-acyl-L-homoserine lactones (AHLs) molecules produced by other bacterial species. Consequently, it consists of two main conserved domains: the autoinducer (AHL) binding domain (residues 24 to 156) and the DNA binding domain (residues 180 to 240) [45]. The results of docking SdiA with eugenol and eugenol acetate showed that these molecules bind to two distinct domains with different binding energies. Indeed, the complex formed by eugenol acetate was more stable (−5.5 kcal/mol) compared to that of eugenol (−4.3 kcal/mol), which could be explained by the presence of the acetate group, which makes the molecule less polar and therefore better suited to the hydrophobic pocket of the binding site, promoting additional stabilizing interactions [46]. This trend was also observed among TEO compounds, where *p*-cymene, the most hydrophobic molecule, showed the strongest affinity, followed by thymol, while the more polar linalool displayed the lowest binding energy. Similarly, *α*-pinene and eucalyptol, the major constituents of EEO, exhibited favorable binding energies consistent with their hydrophobic nature. These results could confirm those reported by Adeosun et al. [45] which have demonstrated that phytochemical molecules such as phytol, camphene, and *α*-pinene can bind to SdiA and significantly reduce the expression of virulence factors. Interestingly, despite the theoretical affinities of the EEO compounds, the EEO showed the lowest anti-adhesion activity in vitro. This discrepancy suggests that strong binding to a single quorum-sensing regulator may not directly translate into a significant antibiofilm effect, as biofilm formation is a multifactorial process involving multiple regulatory pathways and structural determinants [47]. Furthermore, these findings highlight that the biological activity of an essential oil results from the combined contribution of its various constituents rather than solely from its major compounds [48]. The analysis of the amino acids involved in the interactions revealed that the residues bound by eugenol (Lys216, Gln219, Lys220, Asn223, and Ala224) are part of the DNA-binding domain, while the other tested EOs compounds interacted with residues of the autoinducer binding site (Cys45, Val57, Phe59, Tyr63, Trp67, Tyr71, Gln72, Asn75, Leu77, Ala109, Ala110 and His113). This duality of targets suggests the possibility of a complementary or synergistic effect between the molecules in inhibiting SdiA.

MrkD is the terminal adhesive subunit of type 3 fimbriae in *K. pneumoniae*. It plays a crucial role in bacterial adhesion to both abiotic surfaces and host cells, representing a critical step in the initiation of biofilm formation. Structurally, MrkD consists of two distinct functional domains: a lectin domain, located at the N-terminal position (residues 24 to 184), responsible for the specific recognition of substrates such as collagen (present in the interstitial connective tissues of the kidney), and a pilin domain at the C-terminal position (residues 185 to 332), which allows the adhesin to anchor to the fimbriae shaft [9,49]. Molecular docking of this protein with the major compounds of tested EOs revealed increasing affinity in the following order: eucalyptol (−5.3 kcal/mol), eugenol (−5.4 kcal/mol), linalool (−5.5 kcal/mol), eugenol acetate (−5.7 kcal/mol), *α*-pinene (−6.0 kcal/mol), *p*-cymene (−6.4 kcal/mol), and thymol (−7.0 kcal/mol). These findings suggest that thymol benefits from a balanced interaction profile, allowing both polar anchoring and efficient hydrophobic packing within the binding site. This structural complementarity likely accounts for its enhanced binding stability compared to the other tested EOs constituents [50]. These observations are consistent with the study of Yuan et al. [51], which showed that thymol, in particular, could eliminate a mature MRSA biofilm by disrupting the matrix cohesion of the biofilm. The amino acids involved in the bonds of these compounds are all part of the lectin domain, suggesting that the eradicating action of this oil is based on the ability of these compounds to induce local destabilization of the lectin site and/or disrupt interactions already formed between MrkD and its surface substrates.

Overall, monoterpene hydrocarbons (*p*-cymene and *α*-pinene) showed superior binding performance, likely due to their hydrophobic compatibility with the target binding pockets. Oxygenated monoterpenes (linalool and eucalyptol) exhibited more variable affinities, whereas phenolic compounds (thymol and eugenol derivatives) demonstrated balanced interaction patterns involving both aromatic stabilization and potential polar contacts. However, protein–ligand affinity depends not only on ligand structure but also on the physicochemical characteristics and spatial configuration of each binding pocket, including residue composition, polarity, steric constraints, and flexibility. Docking interactions, therefore, remain strongly target-dependent, and high affinity toward one regulator cannot be directly extrapolated to other proteins with distinct structural environments. Moreover, although molecular docking predicts potential binding stability, biofilm inhibition is a multifactorial and systems-level process; thus, in silico affinity values cannot be directly translated into in vitro efficacy.

Our findings highlight the multi-target complexity of antibiofilm mechanisms and suggest that the biological activity of essential oils likely results from cumulative, potentially synergistic modulation of multiple regulatory pathways rather than selective inhibition of a single protein. Nevertheless, certain limitations must be acknowledged, including the relatively limited number of bacterial strains tested, which restricts the generalizability of the results, and the absence of functional validation confirming the direct involvement of the investigated targets in biofilm formation. Future studies are further required to strengthen and validate the proposed mechanisms.

## 4. Materials and Methods

### 4.1. Plant Collection and EOs Extraction

The two plant species, *T. hirtus* Willd. Ssp. *algeriensis* and *E. globulus*, were collected in the localities of Aghrib (36°48′08″ N, 4°19′22″ E) and Yakouren (36°44′05″ N, 4°26′19″ E) in the Tizi-Ouzou region, respectively. To ensure batch standardization, aerial parts were sampled from multiple plants, at the same phenological stage, and from all four cardinal directions of the collection site. On the other hand, *S. aromaticum* was procured from a local herbalist in Tizi-Ouzou. All collected material was pooled to obtain a homogeneous sample prior to EOs extraction.

The essential oils were extracted using the steam distillation method and characterized by GC-MS, as previously reported by Bariz et al. [18], which provides the complete qualitative and quantitative composition. The composition of *T. hirtus* Willd. Ssp. *algeriensis* EO was predominantly thymol (23.14%), followed by linalool (18.44%) and *p*-cymene (16.17%). The composition of *E. globulus* EO was mainly eucalyptol (54.29%) and *α*-pinene (17.32%). The *S. aromaticum* EO showed a composition rich mainly in eugenol (80.46%) and eugenol acetate (16.23%). The present in silico study was conducted based on the compounds identified in this previously characterized batch.

### 4.2. Tested Bacterial Strains

Five strains of *K. pneumoniae* were tested in this study, including one reference strain, *K. pneumoniae* ATCC 700603 ESBL (SHV-18)-positive, and four MDR clinical strains producing CTX-M-type ESBLs: *K. pneumoniae* 3520, *K. pneumoniae* 1216, *K. pneumoniae* 5111, and *K. pneumoniae* 3511, identified by MALDI-TOF. These strains were evaluated for their sensitivity to our three essential oils using the microdilution method, according to the CLSI M07-A9 [52].

### 4.3. Biofilm Formation Test

The ability of *K. pneumoniae* strains to form biofilms was evaluated on 96-wellmicroplates, as described by Saoudi et al. [53]. A standard bacterial suspension was used to prepare a culture of TSB supplemented with 2% glucose (TSBG) at a concentration of 10^6^ CFU/mL. 100 μL of this bacterial culture was then mixed with 100 μL of TSBG in each well. After incubation of 48 h at 37 °C, the contents of the wells were discarded and the microplate was washed twice in succession with saline water (0.9%) to remove all the planktonic cells. After washing, the microplate was dried at 60 °C for 1 h. The preformed biofilm at the bottom of the wells was then stained with 150 μL of 1% crystal violet for 15 min. Subsequently, the crystal violet was removed, and the microplate was rinsed three times with sterile water. Finally, 150 μL of methanol (99%) was added to each well for 15 min. All assays were conducted in triplicate. The optical density (OD) was then recorded at 630 nm using an ELISA plate reader (Micro Lab Instruments, Ahmedabad, India). Uninoculated TSB served as the negative control, and the cut-off value was determined according to the following formula [54]: ODc = Average OD of negative control + 3 SD (Standard Deviation) of negative control.

Based on the cut-off OD calculated, strains were classified into the following categories: Non-biofilm producers (OD < ODc), weak biofilm producers (ODc < OD < 2 × ODc), moderate biofilm producers (2 × ODc < OD < 4 × ODc), and strong biofilm producers (OD > 4 × ODc).

### 4.4. Anti-Adhesion Activity of Essential Oils

The anti-adhesion activity of biofilm-forming strains was evaluated on a 96-well microplate. The wells were filled with 100 µL of essential oil dissolved in TSBG, then 100 µL of the bacterial culture (10^6^ CFU/mL) prepared in TSBG was added. The final concentration of essential oil in the well was equivalent to the MIC. The microplates were then incubated for 24 h at 37 °C, and the biofilm was quantified using crystal violet staining as previously described. All tests were performed in triplicate. The percentage of adhesion inhibition was calculated according to the following formula [55]:

$$\%\;of\;inhibition=\frac{\left(OD\;growth\;control)-(OD\;sample\right)}{OD\;(growth\;control)}\times 100$$

### 4.5. Eradication Activity of Essential Oils

The ability of essential oils to eradicate preformed biofilms was evaluated as follows: the biofilm was first formed over 48 h on the microplate. The wells were washed three times with sterile physiological saline. 200 µL of essential oil was then added to each well at a concentration equal to the MIC. After incubation for 24 h at 37 °C, the biofilm was quantified using the crystal violet method. Wells containing 100 µL of sterile distilled water served as controls. All tests were performed in triplicate. The percentage of biofilm inhibition or eradication was calculated by comparing the absorbance of the untreated biofilm with that of the treated biofilm using the following formula [56]:

$$\%\;of\;eradication=\frac{\left(OD\;growth\;control)-(OD\;sample\right)}{OD\;(growth\;control)}\times 100$$

### 4.6. Scanning Electron Microscope (SEM)

The eradicating effect of biofilms was observed under an electron microscope as follows: 24-h biofilms were first formed on glass slides as described above. After incubation, the biofilms were treated with essential oil at a concentration corresponding to the MIC for 60 min. The slides were then washed with sterile PBS, and the residual biofilm was fixed with glutaraldehyde (2.5% *v*/*v*) for 4 h at 4 °C. After fixation, the slides were dehydrated with ethanol at increasing concentrations (30%, 40%, 50%, 60%, 70%, 80%, 90% *v*/*v*) for 20 min. The slides were then dried overnight at room temperature before observation under a scanning electron microscope (CIQTEK SEM5000X, Hefei, China) [57].

### 4.7. Molecular Docking

Based on the results of the antibiofilm activity tests, it was hypothesized that the activity of the tested EOs could be attributed to the ability of their major compounds to interact with proteins involved in biofilm formation. Therefore, molecular docking was performed to define the types of bonds formed by these molecules with bacterial targets. Three proteins were targeted: two transcriptional regulators, MrkH and SdiA, and one adhesin, MrkD. The crystal structures of these proteins were retrieved from the Protein Data Bank (https://www.rcsb.org/ (accessed on 15 April 2025)) with the respective PDB codes 5EJL, 4LFU, and 3U4K. These structures were then prepared by removing water molecules and co-crystallized ligands using Discovery Studio Visualizer 2021. The active site of 5EJL, 4LFU was determined using AutoDockTools software-1.5.6, based on previous bibliographic reports [39,45,49], while 3U4K was subjected to blind docking. On the other hand, the 2D chemical structures of the main compounds of EOs (eugenol, eugenol acetate, thymol, *p*-cymene, linalool, eucalyptol and *α*-pinene) were extracted from the PubChem database. ChemBio3D 16.0 was then used to establish their 3D structures and energy minimization. Finally, the protein and ligand files were converted using Auto Dock 4.2 (MGL Tools 1.5.6) to the required format before running the docking in AutoDock Vina. Nine conformations of each ligand were studied during this procedure, and the best conformation (having the lowest free binding energy) was visualized and analyzed with DS Visualizer 2021. In order to validate the docking procedure, the ligands co-crystallized in the binding sites were re-docked, and the RMSD values were calculated (an RMSD value ≤ 2.0 Å indicates successful validation).

### 4.8. Statistical Analysis

All in vitro experiments were performed in triplicate. The antibiofilm activity of essential oils was analyzed with a one-way analysis of variance (ANOVA) using SPSS 25 with a statistical significance level of 5%.

## 5. Conclusions

The present study showed that the three tested EOs, notably TEO and SEO, were able to inhibit biofilm formation and eradicate preformed biofilms in *K. pneumoniae*. In addition, molecular docking analysis revealed that the main compounds in these EOs interacted in a targeted manner with the MrkH, SdiA, and MrkD proteins, identifying SdiA as the most promising molecular target. These findings suggest a multiple mechanism of action, ranging from disruption of intracellular signaling to direct alteration of adhesion structures. These data thus reinforce the idea that certain EOs could be promising therapeutic alternatives through their multi-target mode of action, for controlling multidrug-resistant biofilms by targeting pathways different from those of conventional antibiotics.

## Figures and Tables

**Table 1:** Biofilm-producing ability of the tested *K. pneumoniae* strains.

Bacterial Strains	OD630 ± SD	Biofilm Formation
*K. pneumoniae* 1216	0.103 ± 0.005	Weak biofilm producer
*K. pneumoniae* 3511	1.131 ± 0.058	Strong biofilm producer
*K. pneumoniae* 3520	0.905 ± 0.128	Strong biofilm producer
*K. pneumoniae* 5111	0.292 ± 0.021	Moderate biofilm producer
*K. pneumoniae* ATCC 700603	0.934 ± 0.142	Strong biofilm producer

**Table 2:** Interactions of the major EOs compoundswith MrkH protein.

Compounds	Score (Kcal/mol)	BindedAminoacids	Bond Types
Eugenol	−5.7	SER140	Hydrogen Bond
		ARG111	Electrostatic
		GLY143; CYS144; LYS182	Hydrophobic
		ALA145	Van der Waals
Eugenol acetate	−5.5	ARG111; SER140; CYS204	Hydrogen Bond
		ARG111	Electrostatic
		LYS182	Hydrophobic
Thymol	−5.0	LEU221; PRO110; ILE225; LEU-6; PRO-4; LEU222	Hydrophobic
*p*-cymene	−5.3	ASP141	Electrostatic
		PRO110; LEU221; ILE225; LEU-6; PRO-4; LEU222	Hydrophobic
Linalool	−4.9	ASP141; GLU218	Hydrogen Bond
		PRO-4; LEU222; ILE225; LEU-6	Hydrophobic
Eucalyptol	−4.7	ARG107; ARG108; ARG109; ARG111; SER140; GLY142; GLY143; LYS182; CYS204; GLN205	Van der Waals
*α*-pinene	−4.6	LYS219; ILE220; TYR172	Hydrophobic

**Table 3:** Interactions of the major EOs compounds with the SdiA protein.

Compounds	Score (Kcal/mol)	BindedAminoacids	Bond Types
Eugenol	−4.3	ASN223; GLN219; ALA224	Hydrogen Bond
		GLN219; LYS216; LYS220	Hydrophobic
Eugenol acetate	−5.5	ASN75; VAL57	Hydrogen Bond
		LEU77; PHE59; TYR71; VAL57	Hydrophobic
Thymol	−5.7	LEU77; PHE59; TYR71; VAL57	Hydrophobic
*p*-cymene	−7.7	CYS45	Pi-Sulfur
		PHE59; VAL68; VAL57; LEU77; PHE59; TYR63	Hydrophobic
Linalool	−4.2	ASN75; GLN72	Hydrogen Bond
		PHE59; TYR71	Hydrophobic
Eucalyptol	−7.1	ALA110; TYR63; TRP67; TYR71	Hydrophobic
*α*-pinene	−7.5	HIS113; ALA109; ALA110; TYR63; TRP67	Hydrophobic

**Table 4:** Interactions of the major EOs compounds with MrkD protein.

Compounds	Score (Kcal/mol)	BindedAminoacids	Bond Types
Eugenol	−5.4	TYR117; LEU73; PHE124	Hydrophobic
Eugenol acetate	−5.7	ARG125; SER123	Hydrogen Bond
		THR119; VAL65; LEU73; PRO157; PHE124	Hydrophobic
Thymol	−7.0	SER 129	Hydrogen Bond
		TYR 29; LEU 126; TYR 64; LEU 162	Hydrophobic
*p*-cymene	−6.4	PHE 124; ILE 109; LEU 126	Hydrophobic
Linalool	−5.5	SER75	Hydrogen Bond
		VAL65; LEU73; PRO157; PHE124	Hydrophobic
Eucalyptol	−5.3	TYR117	Hydrophobic
*α*-pinene	−6.0	PHE124	Hydrophobic

## Data Availability

The data sets during and/or analyzed during the current study are available from the corresponding author upon reasonable request.

## Abbreviations

The following abbreviations are used in this manuscript:

AHL	N-acyl-L-homoserine lactones
agrBDCA	Accessory Gene Regulator system (genes B, D, C, A)
ATCC	American Type Culture Collection
Bla	Betalactamase
agrBDCA	accessory gene regulator BDCA
c-di-GMP	Cyclic di-Guanosine Monophosphate
CFU	Colony-forming unit
CLSI	Clinical and Laboratory Standards Institute
Csu	Chaperone-Usher pili assembly system
CTX-M	Cefotaximase-munich
DNA	Deoxyribonucleic acid
EEO	*Eucalyptus globulus* Essential Oil
EPS	Exopolymeric matrix
ESBL	Extended-spectrum beta-Lactamases
ESKAPEE	*Enterococcus faecium*, *Staphylococcus aureus*, *K. Pneumoniae*, *Acinetobacter baumannii*, *Pseudomonas aeruginosa*, *Enterobacter* spp., and *Escherichia coli*
fimA	Fimbrial protein A
KPC	Klebsiella pneumonia carbapenemase
MALDI-TOF	Matrix-Assisted Laser Desorption/Ionization—Time of Flight
MBL	Metallo-beta-lactamases
MDR	Multi-Drug resistant
MIC	Minimum inhibitory concentration
Mrk D	Mannose-resistant *Klebsiella*-like D
Mrk H	Mannose-resistant *Klebsiella*-like H
NDM	New Delhimetallo-beta-lactamase
PBS	Phosphate-buffered saline
PDB	Protein Data Bank
pqsA	Pseudomonas quinolone signal A
QS	Quorum sensing
RMSD	Root mean square deviation
SdiA	Suppressor of cell division A
SEO	*Syzygium aromaticum* Essential Oil
SHV	Sulfhydryl variable
TEM	Temoniera
TEO	*Thymus hirtus* Willd. Ssp. *algeriensis* Boiss essential oil
TSBG	Tryptic Soy Broth with 2% Glucose
WHO	World Health Organization
